# Conceptualizing Scaffold Guided Breast Tissue Regeneration in a Preclinical Large Animal Model

**DOI:** 10.3390/bioengineering11060593

**Published:** 2024-06-10

**Authors:** Matthew Cheng, Jan Janzekovic, Ronja Finze, Mina Mohseni, Siamak Saifzadeh, Flavia M. Savi, Owen Ung, Michael Wagels, Dietmar W. Hutmacher

**Affiliations:** 1 Centre for Regenerative Medicine, Q Block—Institute of Health and Biomedical Innovation, Queensland University of Technology, 60 Musk Avenue, Kelvin Grove, Brisbane, QLD 4059, Australia; matthew.cheng@hdr.qut.edu.au (M.C.); jnjnzkvc@gmail.com (J.J.); ronja.finze@hdr.qut.edu.au (R.F.); m.mohseni@qut.edu.au (M.M.); siamak.saifzadeh@qut.edu.au (S.S.); flavia.medeirossavi@qut.edu.au (F.M.S.); michael.wagels@health.qld.gov.au (M.W.); 2 Plastic and Reconstructive Surgery, Princess Alexandra Hospital, 199 Ipswich Road, Woollongabba, Brisbane, QLD 4102, Australia; 3 Department of Hand-, Plastic and Reconstructive Surgery, Burn Center, BG Trauma Center Ludwigshafen, University of Heidelberg, 67071 Ludwigshafen, Germany; 4 Medical Engineering Research Facility, Queensland University of Technology, Staib Road, Chermside, Brisbane, QLD 4032, Australia; 5 Breast and Endocrine Surgery, Royal Brisbane and Women’s Hospital, Butterfield St, Herston, Brisbane, QLD 4029, Australia; owen.ung@health.qld.gov.au; 6 Herston Biofabrication Institute, Royal Brisbane and Women’s Hospital, Level 12 Block 7, Cnr Butterfield St & Bowen Bridge Rd, Herston, Brisbane, QLD 4029, Australia

**Keywords:** tissue engineering, regenerative medicine, breast reconstruction, scaffold, polycaprolactone, soft tissue, adipose tissue, pre-clinical, large animal, pig

## Abstract

Scaffold-guided breast tissue regeneration (SGBTR) can transform both reconstructive and cosmetic breast surgery. Implant-based surgery is the most common method. However, there are inherent limitations, as it involves replacement of tissue rather than regeneration. Regenerating autologous soft tissue has the potential to provide a more like-for-like reconstruction with minimal morbidity. Our SGBTR approach regenerates soft tissue by implanting additively manufactured bioresorbable scaffolds filled with autologous fat graft. A pre-clinical large animal study was conducted by implanting 100 mL breast scaffolds (*n* = 55) made from medical-grade polycaprolactone into 11 minipigs for 12 months. Various treatment groups were investigated where immediate or delayed autologous fat graft, as well as platelet rich plasma, were added to the scaffolds. Computed tomography and magnetic resonance imaging were performed on explanted scaffolds to determine the volume and distribution of the regenerated tissue. Histological analysis was performed to confirm the tissue type. At 12 months, we were able to regenerate and sustain a mean soft tissue volume of 60.9 ± 4.5 mL (95% CI) across all treatment groups. There was no evidence of capsule formation. There were no immediate or long-term post-operative complications. In conclusion, we were able to regenerate clinically relevant soft tissue volumes utilizing SGBTR in a pre-clinical large animal model.

## 1. Introduction

Breast cancer is the most common cancer globally [1]. Soft tissue reconstruction following breast cancer treatment is an important consideration, as there is good evidence that it improves physical and psychological well-being [2,3]. The most common method of breast reconstruction is the use of silicone implants [4]. Silicone implants have been developed as a permanent prosthesis. However, this stimulates a foreign-body response resulting in fibrous capsule formation, which can lead to capsular contracture, causing breast deformities, pain, and device failure [5,6]. This is a significant drawback which essentially limits the lifespan of breast implants, often resulting in revision surgery [7,8] or conversion to autologous reconstruction [9].

Autologous reconstruction with free tissue transfer offers a robust method for breast reconstruction. The most common method is an abdominal-based free flap on the deep inferior epigastric artery. These flaps have higher satisfaction rates compared to silicone implants, likely because they offer a more like-for-like reconstruction, highlighting the advantage of autologous tissue [10,11]. However, these flaps require patients to have an adequate donor site for free flap harvest, and there are associated donor site morbidities such as abdominal bulge or hernia [12]. Additionally, free tissue transfer is complex and the surgery requires a high level of specialized care, which can limit patients’ access to this type of surgery. Autologous fat graft is an alternative method for breast reconstruction which is safe and minimizes the donor site requirement [13,14]. However, the variable rate of fat graft survival usually means it is limited to small volume correction or repeat procedures to reconstruct large volumes [15]. Therefore, there are limitations to the current methods of breast reconstruction, largely because these techniques replace tissue rather than regenerating it.

A recent review explores the perspectives offered by bioengineering to regenerate soft tissue, which holds immense promise in the field of breast reconstruction [16]. One approach to stimulating the regeneration of soft tissue utilizes additively manufactured chambers with a pedicled fat flap [17,18]. However, the long-term outcomes are not reported, and there has been variable success. Another approach has used folded surgical mesh [19] at the time of implantation; however, this technique has not been successfully reproduced by others, likely due to is complexity [20].

**Figure 1:**
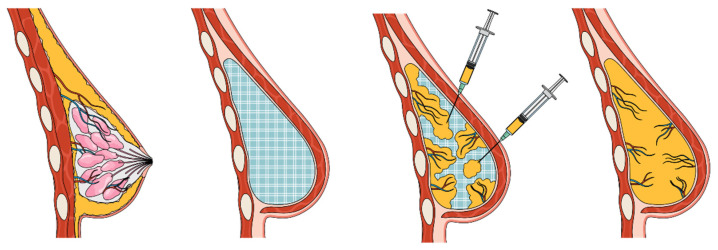
Scaffold-guided breast tissue regeneration concept. Patient-specific breast scaffolds are additively manufactured from medical-grade polycaprolactone and implanted in the breast. Scaffolds are filled with autologous fat graft. The body acts as a bioreactor which initiates and guides soft tissue regeneration. Eventually, the scaffold completely resorbs, resulting in a construct of regenerated soft tissue which has a specified shape and volume.

Scaffold-guided breast tissue regeneration (SGBTR) utilizes additively manufactured, bioresorbable breast scaffolds made from medical-grade polycaprolactone (mPCL), which are filled with autologous fat graft. Over time, the body acts as a bioreactor, which supports the regeneration of soft tissue whilst the scaffold dissolves, resulting in a tissue-engineered construct of regenerated soft tissue (Figure 1). The mPCL biomaterial has a long history of being safely used in Food and Drug Administration (FDA)-approved and Conformite Europeenne (CE)-marked implantable devices and sutures [21]. It is highly biocompatible and completely resorbs through hydrolysis [22].

Additive manufacturing techniques using mPCL can produce patient-specific implants which can have complex porous geometries with high accuracy and reproducibility. Reproducible control of the internal scaffold architecture is essential as the internal structure plays a critical role in guiding cell infiltration, tissue organization, and ultimately homeostasis [23]. While extensive control of scaffold design is currently possible, rational design that directs cell organization to achieve specific tissue structures is not yet a commonly taken approach and remains a challenge. In addition, optimal design relies on knowledge of how cells sense and respond to different scaffold morphologies, compositions, and surface properties during the regeneration process, which is still largely unknown.

We hypothesize that using bioresorbable mPCL scaffolds which are additively manufactured will result in a clinically acceptable and reproducible method to regenerate soft tissue. The aim of this study is to describe the long-term tissue regeneration outcomes and mechanical properties of SGBTR in a preclinical large animal model.

## 2. Materials and Methods

### 2.1. Scaffold Design, Manufacturing, and Characterization

Scaffolds were designed to be 100 mL, approximating the shape and size of a woman’s A-cup-sized breast. The same design was used for all implanted scaffolds. The scaffolds were additively manufactured from medical-grade polycaprolactone (mPCL). An 8 mm pore size and 90° layer rotation on every alternative layer were used. Scaffolds had 90% porosity and 100% pore connectivity, and each strut was 350 µm in diameter. Scaffolds were pre-treated with plasma using a Harrick Plasma Cleaner (Harrick, New York, NY, USA) and sterilized by gamma radiation using an external company which conformed to ISO 11137 [24] (Steritech Pty. Ltd., Brisbane, Australia).

### 2.2. Large Animal Study

A detailed methodology on the animal model used has been published [25]. All animal studies were approved by the Queensland University of Technology (QUT) University Animal Ethics Committee (Approval Number: 1600000282) and in accordance with the requirements on the Animal Code for the Care and Use of Animals for Scientific Purposes (8th Ed, 2013).

**Figure 2:**
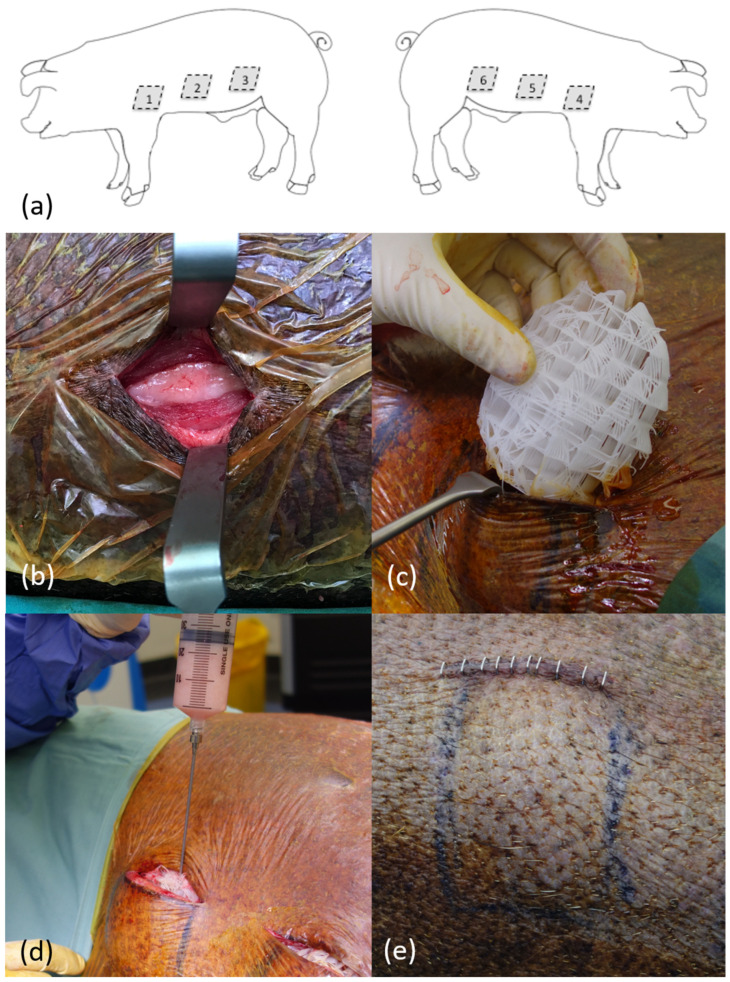
Surgical procedure implanting medical-grade polycaprolactone (mPCL) breast scaffolds. (**a**) Schematic showing treatment site (1–6) placement on flank of pig. (**b**) Pocket development for mPCL scaffold implant under panniculus carnosus muscle layer. (**c**) Porous 100 mL mPCL breast scaffold being implanted in pocket. (**d**) Implanted mPCL scaffold being filled with autologous fat graft. (**e**) Implanted scaffold following wound closure along flank of pig, showing projection.

A total of 55 scaffolds, which were 100 mL in volume, were implanted in 11 adult female immunocompetent Australian minipigs under general anesthesia (Figure 2). These scaffolds were implanted in the flanks of the pigs under their superficial muscle (panniculus carnosus) layers. Six treatments were performed on each animal, with treatments randomized to their site. Some treatment sites required a second procedure four weeks later to fill the scaffold with autologous fat graft in a delayed fashion.

The treatment groups were:

1. 50 mL fat graft injection only, no scaffold implanted (control);
2. Scaffold only implanted (control);
3. Scaffold implanted and filled with 50 mL immediate fat graft;
4. Scaffold implanted and filled with 50 mL immediate platelet-rich plasma;
5. Scaffold implanted and filled with 50 mL delayed fat graft after four weeks;
6. Scaffold implanted and filled with 50 mL immediate platelet-rich plasma, then 50 mL delayed fat graft after four weeks.

Autologous fat grafts were harvested from the animals’ abdomens using a Body-Jet Eco (Human Med AG, Schwerin, Germany) liposuction system. Platelet-rich plasma (PRP) was harvested from the animals’ whole blood using the Angel (Arthrex, Naples, FL, USA) system and mixed with platelet-poor plasma and 10 mL of 10% calcium chloride for a final 50 mL volume. Animals were reviewed daily for eight weeks whilst housed in an animal facility with veterinary support. They were then transferred to an agistment facility and regularly monitored until a 12-month endpoint, when they were euthanized to facilitate post-humous analysis.

### 2.3. Post-Humous Analysis

Imaging was performed with ultrasound (US), computed tomography (CT), and magnetic resonance imaging (MRI) to determine the volume and distribution of regenerated tissue. Immediately after euthanasia, a CT scan (Toshiba Aquilion Lightning, Tokyo, Japan) using standard helical acquisition was performed. Scaffolds were then explanted. At the fat-graft-only treatment site, a portion of tissue including skin, subcutaneous fat, panniculus carnosus muscle, and deep muscle was excised within the boundaries marked by tattoo at time of initial surgery. A repeat CT was performed on each explanted sample with 0.5 mm slice thickness. Each sample was then scanned using a 3T-MRI (Siemens MAGNETOM Prisma, Munich, Germany) using T1 and T2 fat suppression sequences, as well as wavelength excitation curves to investigate water and fat peaks.

CT volumetric analysis was performed using a free open-source medical image reader (Horos 4.0.1). The total volume of regenerated tissue in the explanted scaffold was calculated by segmenting a region of interest (ROI) of greater than −200 Hounsfield units to capture all tissue other than air. This calculated volume was correlated with a water displacement technique using the explanted samples. The volume of fat was calculated by segmenting a ROI of −50 to −200 Hounsfield units, which corresponds to the density of fat in pigs [26].

Distribution analysis was performed using a free open-source image processor (ImageJ 1.54i). Fat saturated sequences were used, and a middle slice through the scaffold construct was imported. This slice was divided into three zones and converted to a binary scale. The proportion of high signal, corresponding to fat, and low signal, corresponding to other tissue types, was calculated in each zone.

Histological and immunohistochemistry studies were performed to analyze the structure and composition of the regenerate tissue. Slides were stained with hematoxylin and eosin (H&E) for general analysis. Immunohistochemistry was performed using Perilipin-1 (PLN1) (Anti-Perilipin-1 antibody, ab3526, Abcam, Cambridge, UK) antibodies, which have been used as markers to identify viable adipocytes [27]; Masson’s trichrome for connective tissue; and von Willebrand Factor (vWF) as a marker for vascularity.

Mechanical studies were performed to determine the mechanical properties of the regenerated soft tissue construct. Explanted samples were tested using an Instron 5848 MicroTester (Instron, Norwood, MA, USA) at a strain rate of 0.1 mm/s. Scaffolds were placed into 37-degree Celsius phosphate-buffered saline (pH = 7) and compressed to 50% to calculate an approximate elastic modulus.

### 2.4. Statistical Analysis

Statistical analysis was performed using SPSS v26 (IBM) and Microsoft Excel (Version 2405 Build 16.0.17628.20006). Significance was defined as *p* < 0.05. Mean values are reported as mean ± standard errors with a 95% confidence internal.

## 3. Results

### 3.1. Macroscopic Analysis

**Figure 3:**
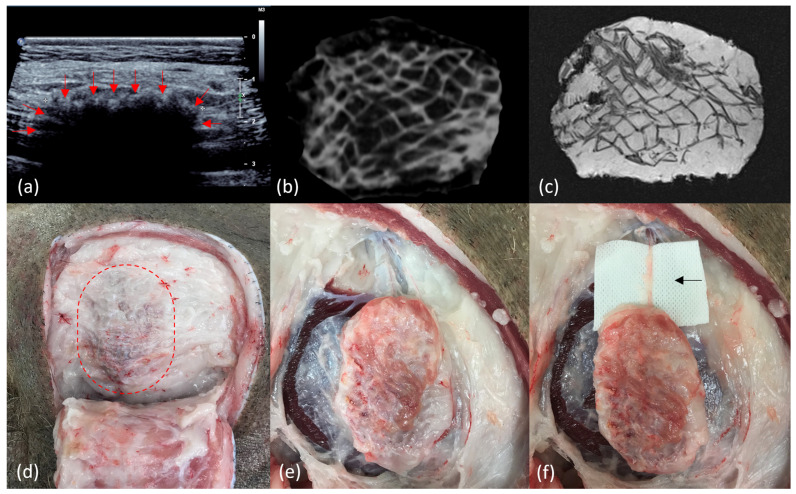
Radiological and macroscopic images of scaffolds at the 12-month end point. (**a**) Ultrasound of scaffold in situ, demonstrating tissue infiltration from host tissue into the scaffold marked by arrows. (**b**) Computed tomography of explanted scaffold demonstrating a hypodense signal of adipose tissue filling the scaffold structure, which has a hyperdense signal. (**c**) Magnetic resonance imaging with fat saturated (T1) sequence of explanted scaffold demonstrating the high signal of adipose tissue filling the scaffold structure, which has a low signal. (**d**) View of scaffold in situ (dotted line) with overlying soft tissue reflected back, demonstrating significant tissue integration and a lack of fibrous capsule formation. (**e**) View of scaffold partially dissected from surrounding tissue, demonstrating a lack of scaffold migration or rotation, significant tissue integration, and a lack of fibrous encapsulation. (**f**) View of completely dissected scaffold filled with soft tissue. There is an arrow showing a dissected blood vessel growing into the scaffold from a major blood vessel in the surrounding host tissue.

The surgical procedure and scaffolds were well tolerated by all animals (*n* = 11) across the 12-month study period (Figure 2). There were no local wounds or systemic complications across any of the treatment sites (*n* = 66). All scaffolds (*n* = 55) were completely filled with soft tissue macroscopically and radiologically (CT and MRI) (Figure 3). A fibrous capsule was not identified around any of the scaffolds (Figure 3). Macroscopically, all scaffolds demonstrated significant tissue integration, and this is supported by ultrasound showing tissue infiltration into the scaffold structure (Figure 3). No scaffolds demonstrated significant rotation or migration from the implantation site. Blood vessels were identified growing into the scaffold structure from major vessels in the surrounding host tissue (Figure 3).

### 3.2. Volume and Distribution

**Figure 4:**
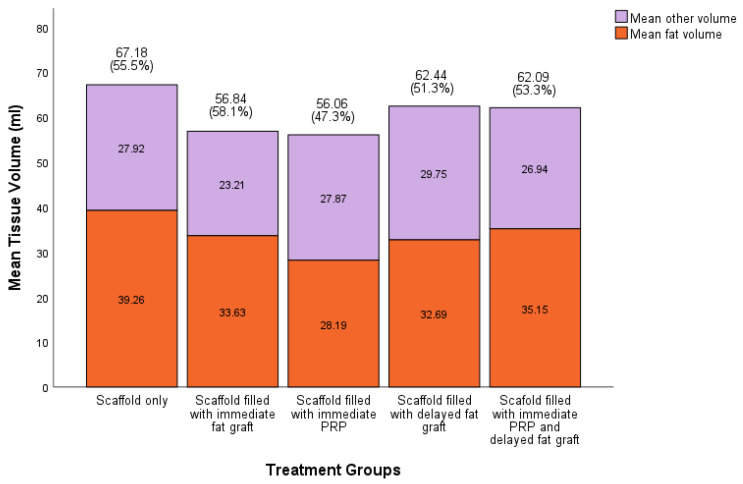
Proportion of fat to other tissue types in scaffolds per treatment group (*n* = 11 in each treatment group). Greatest proportion of fat in scaffold + immediate fat graft group compared to scaffold + immediate platelet-rich plasma (PRP). No statistically significant difference between groups using Kruskal–Wallis H test.

We were able to regenerate and sustain a mean soft tissue volume of 60.9 ± 4.5 mL (95% CI) at 12 months across all scaffolds. There was no statistically significant difference in the total volume of soft tissue sustained between treatment groups (Figure 4). Across all groups, the soft tissue was primarily composed of adipose tissue compared to other soft tissue types, with the highest proportion of adipose tissue regenerated in the scaffolds filled with the immediate fat graft group and the lowest in the scaffold filled with immediate PRP. In scaffolds filled with fat grafts, there was no statistical difference in the volume of soft tissue sustained compared to scaffolds not filled with fat grafts (60.46 ± 5.6 mL (95% CI), 61.62 ± 7.8 mL (95% CI), respectively, *p* > 0.05).

**Figure 5:**
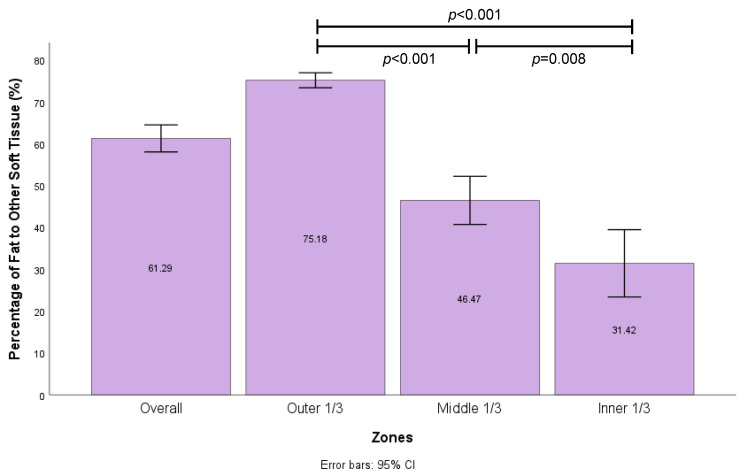
Proportion of fat per zone in each scaffold based on MRI analysis with error bars (*n* = 43 in each group). Significant statistical difference between outer 1/3, middle 1/3, and inner 1/3 zones on Welch’s ANOVA and Games–Howell’s post hoc test.

MRI analysis was performed to study the distribution of soft tissue regeneration throughout the scaffold structure at 12 months (Figure 5). The proportion of fat per zone in each scaffold was based on MRI analysis with error bars (*n* = 43 in each group). A significant statistical difference was found between the outer 1/3, middle 1/3, and inner 1/3 zones on Welch’s ANOVA and Games–Howell’s post hoc test. The largest percentage of adipose tissue was identified in the outer 1/3 (75%) compared to the middle 1/3 (46%) and inner 1/3 (31%) zones of the scaffold, which were all statistically different (*p* < 0.001 and *p* = 0.008).

### 3.3. Microscopic Analysis

**Figure 6:**
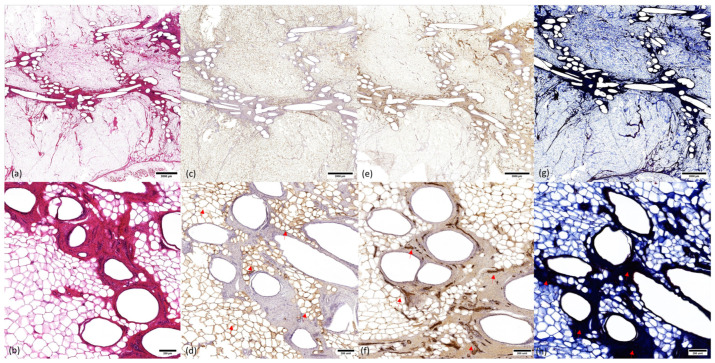
Histology and immunohistochemistry of explanted mPCL scaffolds at 12 months. (**a**) Hematoxylin and eosin (H&E) overview (0.7× zoom). Central arrangement of scaffold struts, adjacent rich extracellular matrix (ECM) network, and regenerated adipose tissue filling the scaffold pores. (**b**) H&E high magnification (5× zoom) showing arrangement of scaffold struts, ECM network, and adipocytes. (**c**) Overview (0.7× zoom) of Perilipin (PLN1) stain demonstrating viable adipocytes (arrows). (**d**) High magnification (5× zoom) view of PLN1 stain demonstrating viable adipocytes. (**e**) Overview (0.7× zoom) of von Willebrand (vWF) stain demonstrating vasculature in the rich ECM network surrounding mPCL struts and around regenerated adipocytes. (**f**) High-magnification (5× zoom) view of vWF stain demonstrating vascularity (arrows). (**g**) Overview (0.7× zoom) of Masson’s trichome stain demonstrates intense collagen staining in the ECM zone surrounding mPCL struts. There is no evidence of fibrous capsules at the periphery of the scaffold. (**h**) High-magnification (5× zoom) view of Masson’s trichrome stain showing ECM network around scaffold struts (arrows).

Tissue structure and composition were assessed with histology and immunohistochemistry (Figure 6). H&E overview demonstrated an arrangement of circular mPCL bars and struts, surrounded by an adjacent rich extracellular matrix (ECM) and an outer zone of regenerate adipocytes. The presence of adipocytes was confirmed using PLN1 stain. There was rich vascularity in the ECM zone and around adipocytes on vWF analysis. Masson’s trichrome demonstrated collagen staining around scaffold struts. There was no evidence of a microscopic confluent fibrous capsule at the periphery of the scaffold.

### 3.4. Mechanical Testing

**Figure 7:**
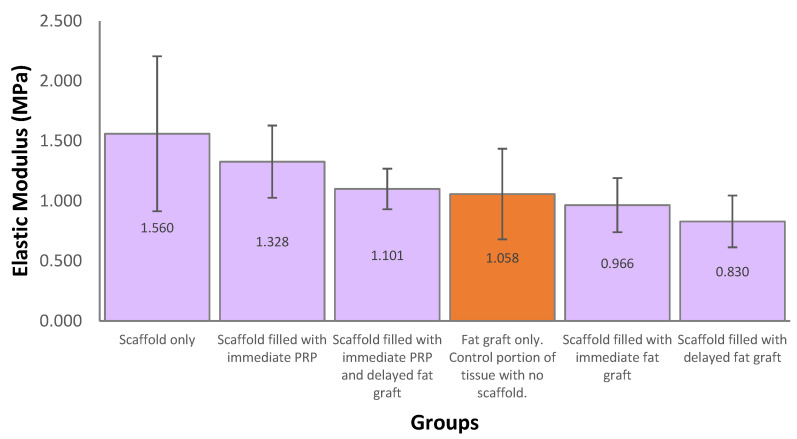
Mechanical testing of explanted samples at 12 months post-implantation. Samples were immersed in a water bath filled with phosphate-buffered saline (pH = 7) at 37 °C. The test was performed with up to 50% uniaxial compression and a strain rate of 0.1 mm/s. *n* = 10 in each group. The orange bar represents a tested control portion of excised soft tissue with no scaffold.

The mean elastic modulus for explanted scaffolds was 1.157 MPa, which was similar to a control portion of tissue excised at the fat-graft-only treatment site, which was 1.058 MPa (Figure 7).

## 4. Discussion

The ability to regenerate soft tissue through SGBTR is an emerging field of research. This study was able to regenerate and sustain a mean soft tissue volume of 60.9 ± 4.5 mL (95% CI) over 12 months. Importantly, in the treatment groups where scaffolds were not filled with autologous fat grafts, this study regenerated 66.1 ± 9.7 mL (95% CI), representing de novo regeneration of soft tissue. In contrast, scaffolds filled with 50 mL of autologous fat graft regenerated 59.5 ± 4.6 mL (95% CI), representing a modest 20% increase in soft tissue, but this did not account for potential fat graft resorption, which may increase that gain. In other large animal studies, Shim et al. [28] were also able to regenerate de-novo soft tissue utilizing empty PCL scaffolds designed in a ball-like shape in four pigs over three months. However, their scaffolds were significantly smaller (3 cm in diameter equating to 14 mL volume) compared to our volumes (100 mL), limiting their clinical applicability. Chhaya et al. [29] were able to achieve 6.1-fold and 4.95-fold increases in soft tissue in 75 mL mPCL scaffolds which were filled with immediate and delayed fat grafts, respectively, over three months. Our study supports the previous work in terms of achieving soft tissue regeneration utilizing mPCL scaffolds, and to date, it is the largest and longest pre-clinical large animal study highlighting the safety and efficacy of SGBTR.

The composition of regenerated tissue is an important consideration. In the context of breast reconstruction, the ability to regenerate fat is critical, as the breast is predominantly composed of adipose tissue [30]. However, the breast is also composed of other soft tissue, such as glandular and connective tissue [30,31]. In our study, the regenerated soft tissue was largely composed of adipose tissue across all treatment groups. This was confirmed with immunohistochemistry using PLN1. We also regenerated other soft tissue types, including fibrotic and vascular tissue, confirmed with Masson’s trichrome and vWF stains, respectively. The ideal proportion of adipose tissue to other tissue types is largely unknown, but the ability to modulate the type and proportion of regenerated soft tissue will be key for clinical applications.

Chhaya et al. [29] identified the positive influence of autologous fat grafts on increasing adipose tissue regeneration in a pilot study. We similarly identified that the highest proportion of adipose tissue regenerated was in the treatment group filled with immediate fat grafts, but this did not reach statistical significance. In fact, we identified that there was largely no effect of various treatments, such as fat graft, timing, and PRP, on tissue regeneration. The difference may be due to the larger scaffold sizes investigated in our study, meaning a longer distance from the injected fat graft to peripheral vascularized tissue. On MRI analysis, we identified that the greatest proportion of adipose tissue sustained was in the outer 1/3 of the scaffold, closest to the peripheral vascularized tissue, and the least was in the inner 1/3, which was furthest from vascularized tissue. This closely follows a model on fat graft survival by Eto et al. [27], where there is an outer zone of surviving adult adipocytes and adipose-derived stromal cells (ASCs); an intermediate zone of necrotic adipocytes but surviving ASCs, where regeneration occurs; and an inner zone of necrotic adipocytes and ASCs, where no regeneration occurs. This highlights that there may be a use of autologous fat grafts to guide tissue regeneration towards forming more adipose tissue, but vascularity remains a critical consideration.

A different approach to improving adipose tissue vascularization is by transferring vascularized tissue as a fat flap. Findlay et al. [17] investigated a pedicled fat flap in 78.5 mL tissue-engineered chambers to regenerate adipose tissue and achieved a 5-fold increase. This was scaled up in a human trial of five patients [18]. However, there was limited success: One patient formed 210 mL of new tissue, but three patients failed to develop any soft tissue enlargement, and the device was encased in a thick fibrous capsule. Faglin et al. [32] investigated tissue-engineered chambers in rats and pigs and discussed the importance of modulating the fibrous encapsulation around the implants, as it influences the extent of tissue regeneration. This highlights the importance of considering the foreign body response (FBR) to implanted biomaterials and its influence on tissue regeneration.

An FBR is universal to any implanted biomaterial, and usually results in fibrous capsule formation to wall the implant off from the host [33]. In the context of breast reconstruction, a thick, fibrous capsule is undesirable as it can result in capsular contracture, causing breast deformities, pain, and implant rupture [5,6]. We identified no macroscopic evidence of fibrous encapsulation around our scaffolds at 12 months. This is largely due to the porous structure of our scaffolds, which was large enough to encourage tissue integration rather than peripheral fibrous capsule formation [34]. This contrasts with other studies using implants with smaller pores, which have resulted in peripheral fibrous encapsulation [17,18,28]. In addition, we identified that the scaffold FBR resulted in a rich ECM network containing blood vessels, essentially creating an internal vascularized structure around mPCL struts, allowing for tissue regeneration. The use of mPCL is an important factor in creating a favorable FBR for tissue regeneration due to its slow degradation profile. This has led to the ability to regenerate clinically relevant large volumes of soft tissue without vascularized fat flaps.

This study did not cause any significant wound issues or discomfort for the pigs over the 12-month study period, suggesting that mPCL scaffolds are well tolerated. The mechanical properties of the regenerated soft tissue construct are an important factor in this. Mechanical testing on our samples demonstrated a similar elasticity to the soft tissue dissected from the pigs. We also identified that the soft tissue constructs were significantly stiffer than human breast tissue [35]. However, this may be desirable in the context of breast reconstruction to maintain shape and projection over time, negating the effect of gravity and breast ptosis [36,37]. Scaffolds also provide mechanical protection against external forces, which improves fat graft survival [38]. Conscious design and additive manufacturing are critical to engineering reliable and reproducible scaffolds to protect tissue regeneration. It overcomes the repeatability issues reported in [20] when using an alternative method of folding surgical mesh to create a scaffold [19]. An additional benefit of additive manufacturing is the ability to design scaffolds which are patient-matched. Whilst this preclinical study only examined a single breast-shaped scaffold, future application in breast reconstruction would utilize customized scaffolds based on pre-operative imaging of the patient. This has the potential to be applied to wide local excisions/lumpectomies which have unique shapes and allow for more like-for-like reconstruction.

This study did not identify any treatment effects of fat grafting, timing, or PRP on tissue regeneration. This may be due to an inadequate sample size to identify a small effect. The generalizability of this study is limited to smaller clinically relevant volumes approximating an A-cup breast size. The ability to scale this approach to larger volumes may influence the proportion of soft tissue regeneration due to limitations in vascularity. However, this approach has been successfully applied to reconstruct a large-volume pectus excavatum deformity in a human [39].

## 5. Conclusions

This study was able to regenerate clinically relevant volumes of soft tissue in a pre-clinical long-term large animal model utilizing SGBTR principles. This provides safety and efficacy data for future clinical trials.

## Data Availability

Data are not publicly available due to ethical reasons.

## References

[1] Arnold M., Morgan E., Rumgay H., Mafra A., Singh D., Laversanne M., Vignat J., Gralow J.R., Cardoso F., Siesling S. (2022). Current and future burden of breast cancer: Global statistics for 2020 and 2040. Breast.

[2] Rowland J.H., Desmond K.A., Meyerowitz B.E., Belin T.R., Wyatt G.E., Ganz P.A. (2000). Role of breast reconstructive surgery in physical and emotional outcomes among breast cancer survivors. J. Natl. Cancer Inst..

[3] Lovelace D.L., McDaniel L.R., Golden D. (2019). Long-term effects of breast cancer surgery, treatment, and survivor care. J. Midwifery Women’s Health.

[4] Panchal H., Matros E. (2017). Current Trends in Postmastectomy Breast Reconstruction. Plast. Reconstr. Surg..

[5] Headon H., Kasem A., Mokbel K. (2015). Capsular Contracture after Breast Augmentation: An Update for Clinical Practice. Arch. Plast. Surg..

[6] Handel N., Cordray T., Gutierrez J., Jensen J.A. (2006). A long-term study of outcomes, complications, and patient satisfaction with breast implants. Plast. Reconstr. Surg..

[7] Coroneos C.J., Selber J.C., Offodile A.C.I., Butler C.E., Clemens M.W. (2019). US FDA Breast Implant Postapproval Studies: Long-term Outcomes in 99,993 Patients. Ann. Surg..

[8] Finlay B., Kollias V., Hall K.A., Clement Z., Bingham J., Whitfield R., Kollias J., Bochner M. (2021). Long-term outcomes of breast reconstruction and the need for revision surgery. ANZ J. Surg..

[9] Visser N.J., Damen T.H.C., Timman R., Hofer S.O.P., Mureau M.A.M. (2010). Surgical Results, Aesthetic Outcome, and Patient Satisfaction after Microsurgical Autologous Breast Reconstruction following Failed Implant Reconstruction. Plast. Reconstr. Surg..

[10] Eltahir Y., Werners L.L.C.H., Dreise M.M., Zeijlmans van Emmichoven I.A., Werker P.M.N., de Bock G.H. (2015). Which Breast Is the Best? Successful Autologous or Alloplastic Breast Reconstruction: Patient-Reported Quality-of-Life Outcomes. Plast. Reconstr. Surg..

[11] Fracon S., Renzi N., Manara M., Ramella V., Papa G., Arnež Z. (2018). Patient satisfaction after breast reconstruction: Implants vs. autologous tissues. Acta Chir. Plast..

[12] Chang E.I., Chang E.I., Soto-Miranda M.A., Zhang H., Nosrati N., Robb G.L., Chang D.W. (2013). Comprehensive analysis of donor-site morbidity in abdominally based free flap breast reconstruction. Plast. Reconstr. Surg..

[13] Agha R.A., Fowler A.J., Herlin C., Goodacre T.E.E., Orgill D.P. (2015). Use of autologous fat grafting for breast reconstruction: A systematic review with meta-analysis of oncological outcomes. J. Plast. Reconstr. Aesthetic Surg..

[14] Coleman S.R., Saboeiro A.P. (2007). Fat Grafting to the Breast Revisited: Safety and Efficacy. Plast. Reconstr. Surg..

[15] Herly M., Ørholt M., Larsen A., Pipper C.B., Bredgaard R., Gramkow C.S., Katz A.J., Drzewiecki K.T., Vester-Glowinski P.V. (2018). Efficacy of breast reconstruction with fat grafting: A systematic review and meta-analysis. J. Plast. Reconstr. Aesthetic Surg..

[16] Berkane Y., Oubari H., van Dieren L., Charlès L., Lupon E., McCarthy M., Cetrulo C.L., Bertheuil N., Uygun B.E., Smadja D.M. (2024). Tissue engineering strategies for breast reconstruction: A literature review of current advances and future directions. Ann. Transl. Med..

[17] Findlay M.W., Dolderer J.H., Trost N., Craft R.O., Cao Y., Cooper-White J., Stevens G., Morrison W.A. (2011). Tissue-engineered breast reconstruction: Bridging the gap toward large-volume tissue engineering in humans. Plast. Reconstr. Surg..

[18] Morrison W.A., Marre D., Grinsell D., Batty A., Trost N., O’Connor A.J. (2016). Creation of a large adipose tissue construct in humans using a tissue-engineering chamber: A step forward in the clinical application of soft tissue engineering. EBioMedicine.

[19] Rehnke R.D., M Asher Schusterman I., Clarke J.M., Price B.C., Waheed U., Debski R.E., Badylak S.F., Rubin J.P. (2020). Breast reconstruction using a three-dimensional absorbable mesh scaffold and autologous fat grafting: A composite strategy based on tissue-engineering principles. Plast. Reconstr. Surg..

[20] Hwang K., Wu X. (2021). Breast Reconstruction Using a Three-Dimensional Absorbable Mesh Scaffold and Autologous Fat Grafting: A Composite Strategy Based on Tissue-Engineering Principles. Plast. Reconstr. Surg..

[21] Woodruff M.A., Hutmacher D.W. (2010). The return of a forgotten polymer—Polycaprolactone in the 21st century. Prog. Polym. Sci..

[22] Lam C.X., Hutmacher D.W., Schantz J.T., Woodruff M.A., Teoh S.H. (2009). Evaluation of polycaprolactone scaffold degradation for 6 months in vitro and in vivo. J. Biomed. Mater. Res. A.

[23] Mohseni M., Bas O., Castro N.J., Schmutz B., Hutmacher D.W. (2019). Additive biomanufacturing of scaffolds for breast reconstruction. Addit. Manuf..

[24] Sterilization of Health Care Products—Radiation.

[25] Cheng M., Janzekovic J., Mohseni M., Medeiros Savi F., McGovern J., Galloway G., Wong C., Saifzadeh S., Wagels M., Hutmacher D.W. (2021). A preclinical animal model for the study of scaffold-guided breast tissue engineering. Tissue Eng. Part. C Methods.

[26] McEvoy F.J., Madsen M.T., Strathe A.B., Svalastoga E. (2008). Hounsfield Unit dynamics of adipose tissue and non-adipose soft tissues in growing pigs. Res. Vet. Sci..

[27] Eto H., Kato H., Suga H., Aoi N., Doi K., Kuno S., Yoshimura K. (2012). The fate of adipocytes after nonvascularized fat grafting: Evidence of early death and replacement of adipocytes. Plast. Reconstr. Surg..

[28] Shim K.-S., Ryu D.H., Jo H.-S., Kim K.-B., Kim D.-H., Park Y.-K., Heo M., Cho H.-E., Yoon E.-S., Lee W.J. (2023). Breast tissue reconstruction using polycaprolactone ball scaffolds in a partial mastectomy pig model. Tissue Eng. Regen. Med..

[29] Chhaya M.P., Balmayor E.R., Hutmacher D.W., Schantz J.T. (2016). Transformation of Breast Reconstruction via Additive Biomanufacturing. Sci. Rep..

[30] Vandeweyer E., Hertens D. (2002). Quantification of glands and fat in breast tissue: An experimental determination. Ann. Anat.-Anat. Anz..

[31] Gaskin K.M., Peoples G.E., McGhee D.E. (2020). The fibro-adipose structure of the female breast: A dissection study. Clin. Anat..

[32] Faglin P., Gradwohl M., Depoortere C., Germain N., Drucbert A.-S., Brun S., Nahon C., Dekiouk S., Rech A., Azaroual N. (2020). Rationale for the design of 3D-printable bioresorbable tissue-engineering chambers to promote the growth of adipose tissue. Sci. Rep..

[33] Morais J.M., Papadimitrakopoulos F., Burgess D.J. (2010). Biomaterials/tissue interactions: Possible solutions to overcome foreign body response. AAPS J..

[34] Jordan S.W., Fligor J.E., Janes L.E., Dumanian G.A. (2018). Implant porosity and the foreign body response. Plast. Reconstr. Surg..

[35] Ramiao N.G., Martins P.S., Rynkevic R., Fernandes A.A., Barroso M., Santos D.C. (2016). Biomechanical properties of breast tissue, a state-of-the-art review. Biomech. Model. Mechanobiol..

[36] Janzekovic J., Hunt J., Peltz T., Wagels M., Brown T., Hutmacher D.W. (2022). Biomechanical principles of breast implants and current state of research in soft tissue engineering for cosmetic breast augmentation. Aesthetic Plast. Surg..

[37] Rinker B., Veneracion M., Walsh C.P. (2010). Breast ptosis: Causes and cure. Ann. Plast. Surg..

[38] Bao W., Cao L., Wei H., Zhu D., Zhou G., Wang J., Guo S. (2021). Effect of 3D printed polycaprolactone scaffold with a bionic structure on the early stage of fat grafting. Mater. Sci. Eng. C.

[39] Cheng M.E., Janzekovic J., Theile H.J., Rutherford-Heard C., Wille M.-L., Cole C., Lloyd T.B., Theile R.J., Wagels M., Hutmacher D.W. (2022). Pectus excavatum camouflage: A new technique using a tissue engineered scaffold. Eur. J. Plast. Surg..

